# Emerging pneumonia-like illness “legionellosis” in Argentina in the COVID-19 era: Cause to panic?

**DOI:** 10.3389/fphar.2022.1063237

**Published:** 2022-11-08

**Authors:** Ranjan K. Mohapatra, Ahmed Mahal, Venkataramana Kandi, L. V. Simhachalam Kutikuppala, Ashish K. Sarangi, Snehasish Mishra

**Affiliations:** ^1^ Department of Chemistry, Government College of Engineering, Keonjhar, Odisha, India; ^2^ Department of Medical Biochemical Analysis, College of Health Technology, Cihan University—Erbil, Erbil, Kurdistan Region, Iraq; ^3^ Key Laboratory of Plant Resources Conservation and Sustainable Utilization, Guangdong Provincial Key Laboratory of Applied Botany, South China Botanical Garden, Chinese Academy of Sciences, Guangzhou, China; ^4^ Guangzhou HC Pharmaceutical Co., Ltd., Guangzhou, China; ^5^ Department of Microbiology, Prathima Institute of Medical Sciences, Karimnagar, Telangana, India; ^6^ Department of General Surgery, Dr. NTR University of Health Sciences, Vijayawada, Andhra Pradesh, India; ^7^ Department of Chemistry, School of Applied Sciences, Centurion University of Technology and Management, Odisha, India; ^8^ School of Biotechnology, Campus-11, KIIT Deemed-to-be-University, Bhubaneswar, Odisha, India

**Keywords:** legionellosis, epidemiology, complications, transmission route, prevention and control measures

## Introduction

A cluster of eleven cases (seven males and four females of 45 years median age) of legionellosis (severe pneumonia) including four deaths in the Tucuman province of Argentina were reported recently on 3 September 2022 (MPH, 2022a; MPH, 2022b). Legionellosis shows pneumonia-like symptoms, varying from mild febrile to serious illness and sometimes even being fatal. These cases were epidemiologically traced to a private healthcare facility. Out of all the cases, eight were healthcare workers of that facility itself, and three of the four deaths were of these workers. All cases presented similar clinical symptoms like fever, myalgia, bilateral pneumonia, abdominal pain and dyspnea (WHO, 2022). In four cases, *Legionella* sp. was medically identified as the causative organism. Ten cases including the four deaths had underlying comorbidity and severe disease risk (WHO, 2022). As on 3 September 2022, four cases were still hospitalised. Although contacts of these cases are under follow-up surveillance, preliminary investigations revealed no secondary cases, albeit sporadic legionellosis outbreaks earlier are documented in Argentina. Health authorities of the province are coordinating cluster investigation to search for source(s) of infection, identify additional active cases, contact tracing and public health measures to limit further spread.

Initially hospitalised for unrelated reasons, all the eleven reported cases were were shifted to intensive care units after developing pneumonia. Preliminary investigation reports of their blood, respiratory and tissues samples in the local laboratory were negative for respiratory viruses and other suspected bacterial, viral and fungal agents. Samples sent for additional testing to the National Reference Laboratory (the Administration of National Laboratories and Health Institutes; ANLIS) were negative for COVID-19, influenza, hantavirus, *Yersinia pestis*, histoplasma, leptospirosis and a group of 12 respiratory viruses. Additional highly sensitive whole genome sequencing (metagenomics) and bioinformatics analyses of two bronchoalveolar lavage samples revealed similarities with *Legionella* sp. (AMH, 2022). The results of the amplified products of the 16S ribosomal sequences for *Legionella* sp. hinted at similarities with *Legionella pneumophila*, as documented by ANLIS (AMH, 2022). Routine blood culture and serological test were performed to validate the diagnosis of *Legionella* infections. For early and quick diagnosis of suspected *Legionella* case, urine antigen testing and sputum culture are suggested. Urine antigen test is the only tests for *Legionella pneumophila* sero-type whereas sputum culture identifies other serotypes (Brady and Sundareshan, 2022).

Legionellosis by *Legionella* sp. is manifested by pneumonia and related clinical symptoms, typically after 2–10 days incubation although up to 16 days have been recorded in some cases. *Legionella* enters the cell by binding to alveolar macrophages and respiratory epithelial cells, and promotes proliferation by inhibiting the fusion of phagosome and lysosome. Legionellae histopathologic lesions have been noticed in intestinal linings, polymorphonuclear cells and macrophages (Brady and Sundareshan, 2022). With initial mild cough, fever, headache, malaise, loss of appetite and lethargy symptoms, patients could also experience diarrhoea, muscle pain and confusion. Acute respiratory failure, shock, endocarditis, neurological deficits, coma, rhabdomyolysis, renal failure, multiple organ failure, sepsis and death are other complications (CDC, 2022). Usually, if untreated, the disease worsens in the first week. The overall death rate usually is 5%–10% although it may be up to 40%–80% if untreated or if the patient is immunocompromised (WHO, 2022). Medically fit healthy individual exposed to *Legionella* does not fall sick, but individuals of more than 50 years, smokers, the immunocompromised, with chronic lung disease, cancer, diabetes, and kidney or liver failure are at bigger risk (CDC, 2022). This uncommon but important cause of community- and hospital-acquired pneumonia is of public health significance and may cause outbreaks. Rifampin, fluoroquinolones and macrolides are few recommended antibiotics classes, and need to be chosen carefully for effective treatment.

General route of legionellosis transmission is inhaling infective aerosol from contaminated water sources. Infection could occur in vulnerable hospital patients by aspiring contaminated water or ice. As chlorine decomposes at high temperature and *Legionella* is fairly chlorine resistant, hyperchlorination of potable water is futile (Brady and Sundareshan, 2022). Ultraviolet light and copper-silver ionisation unit could be effective against *Legionella* on a sustained basis. No report of direct human-to-human transmission exists yet (WHO, 2022), and legionellosis cases in travellers to Argentina are not reported either.

Legionellae is waterborne and poor water management and the global climate changes could potentially increase the risk of its survival, growth and transmission (Herwaldt and Marra, 2018). The population with respiratory complications like chronic obstructive pulmonary disease are on the rise, attributed to the polluted air that could potentially increase the risk of such individuals to legionellosis (Brady and Sundareshan, 2022). *Legionella* infection could be community-acquired or nosocomial. The corticosteroids and other immunosuppressive therapeutic drugs to alleviate immune reconstitution inflammatory syndrome in the COVID-19 pandemic may also predispose people to legionellosis (Azoulay et al., 2020). In light of this, the predisposed population to legionellosis as post-COVID-19 health complications is rising. The data available in the literature about *Legionella* is limited due to the difficulties in culturing it. Outbreaks in future could be effectively managed through improved water management that includes regular cleaning and maintenance to avoid rusting and biofilm formation, and increased surveillance especially in hospital settings. Air conditioners and water cooling systems may be cleaned and disinfected with biocides to limit microbial growth. There is an urgent need for genomic analysis of the environmental strains of *Legionella* to track and find out virulence genes and assess the associated threats.

Health facilities need to assess risks during healthcare. Health agencies may extend seamless communication strategies to healthcare workers and contiguous community. Global support measures to investigate outbreaks, manage hospitals, sampling, environmental assessment, and infection control and prevention are urgent. Infection prevention and control (IPC) measures may be reinforced and upgraded in the face of COVID-19 pandemic to prevent or reduce healthcare-associated nosocomial transmissions (WHO, 2022). Robust surveillance to locate active and passive cases and isolating them may be done. Environmental sampling, laboratory test and metagenomics to trace and define the source, and implementing effective control measures are highly recommended urgently.

## References

[B1] AMH (2022). Argentina Ministry of Health press release, “ANLIS-MALBRÁN analyzes samples from cases of pneumonia of unknown cause in Tucumán”. Available at: https://www.argentina.gob.ar/noticias/anlis-malbran-analiza-las-muestras-de-los-casos-de-neumonia-de-causa-desconocida-en-tucuman (Accessed September 29, 2022).

[B2] Azoulay E. Russell L. de Louw A. V. Metaxa V. Bauer P. Povoa P. (2020). Diagnosis of severe respiratory infections in immunocompromised patients. Intensive Care Med. 46, 298–314. 10.1007/s00134-019-05906-5 32034433 PMC7080052

[B3] Brady M. F. Sundareshan V. (2022). Legionnaires' disease. [Updated 2022 Jul 4]. In: StatPearls [Internet]. Treasure Island (FL). StatPearls Publishing. Available from: https://www.ncbi.nlm.nih.gov/books/NBK430807/ . 28613558

[B4] CDC (2022). Legionella (legionnaires’ disease and pontiac fiver), march 25, 2021. Available from: https://www.cdc.gov/legionella/about/causes-transmission.html (accessed on 09 29, 22).

[B5] Herwaldt L. A. Marra A. R. (2018). Legionella: A reemerging pathogen. Curr. Opin. Infect. Dis. 31 (4), 325–333. 10.1097/QCO.0000000000000468 29794542

[B6] MPH (2022a). Ministry of public health of the government of tucumán official communication. Available at: https://msptucuman.gov.ar/comunicado-oficial-18/ (Accessed September 29, 2022).

[B7] MPH (2022b). Ministry of public health of the government of tucumán official communication. Available at: https://msptucuman.gov.ar/el-ministerio-de-salud-informo-sobre-la-situacion-sanitaria-del-brote-de-neumonia-bilateral/ (Accessed September 29, 2022).

[B8] WHO (2022). Legionellosis – Argentina, 5 september 2022. Available from: https://www.who.int/emergencies/disease-outbreak-news/item/2022-DON407 (accessed on 09 29, 22).

